# Use of Cardiac MRI in the Diagnosis of Rare Right Ventricular Noncompaction

**DOI:** 10.7759/cureus.14601

**Published:** 2021-04-21

**Authors:** Matthew Montanarella, David Szames, Dheeraj Gopireddy

**Affiliations:** 1 Radiology, University of Florida College of Medicine, Jacksonville, USA

**Keywords:** noncompaction, cardiac mri, right ventricle, cardiomyopathy

## Abstract

Ventricular noncompaction, also previously known as spongy myocardium, is an inherited primary genetic cardiomyopathy. Noncompaction of the left ventricle is seen in the general population typically in the setting of other congenital heart defects and can be a cause of significant morbidity and mortality. Right ventricular noncompaction is a rare form of cardiomyopathy with no definitive diagnostic criteria. Diagnosis of noncompaction of the right ventricle can be concluded using guidance from the diagnostic criteria for left ventricular noncompaction with multi-modality imaging.

## Introduction

Ventricular noncompaction is inherited cardiomyopathy that was previously known as spongy myocardium and was first described in 1926 [1]. This disorder was first classified as primary genetic cardiomyopathy by the American Heart Association in 2008 [2]. Noncompaction refers to the phenomenon of an over trabeculated myocardium with deep intertrabecular recesses communicating with the ventricular cavity [3]. Uncertainty exists regarding the prevalence of this disorder. An estimated prevalence of left ventricular noncompaction in adults referred for echocardiography was estimated to be between 4.5 and 26 per 10,000 patients [4,5]. In a cohort study of primary cardiomyopathies, left ventricular noncompaction was the third most common cause of pediatric cardiomyopathy, at 9.2%, following dilated and hypertrophic cardiomyopathy [6]. A study utilizing an echocardiography database at Texas Children’s Hospital demonstrated a similar percentage of these cases at 9.5% [7].

Noncompaction of the left ventricle can be seen in both sporadic and familial settings and is seen more often in men than women [8]. Noncompaction is typically seen in the setting of other congenital heart defects but can also be seen as an isolated entity [4]. Trabeculation and compaction are essential steps in embryological ventricular development [1]. Noncompaction occurs as a result of the arrest of the normal compaction and most often affects the cardiac apex and mid-ventricular segments [1,9]. This condition is particularly troublesome as it can lead to heart failure, tachyarrhythmia, embolic events, and sudden cardiac death [7,8]. At this time, isolated noncompaction of the right ventricle is a rare phenomenon with only a few case reports discussed in the literature [3,10]. Although no large study data is available for noncompaction of the right ventricle, anecdotally, the case reports seem to involve more men than women. There’s a paucity of data on right ventricular noncompaction and no real prevalence data is available for review. Currently, only diagnostic criteria for noncompaction of the left ventricle exist. The diagnosis of right ventricular noncompaction can be made using a multi-modality approach to imaging using similar diagnostic features of left ventricular noncompaction and by excluding other differential diagnoses. We present a case of right ventricular noncompaction and discuss our methodical and multi-modality approach to diagnosing this rare condition.

## Case presentation

**Figure 1:**
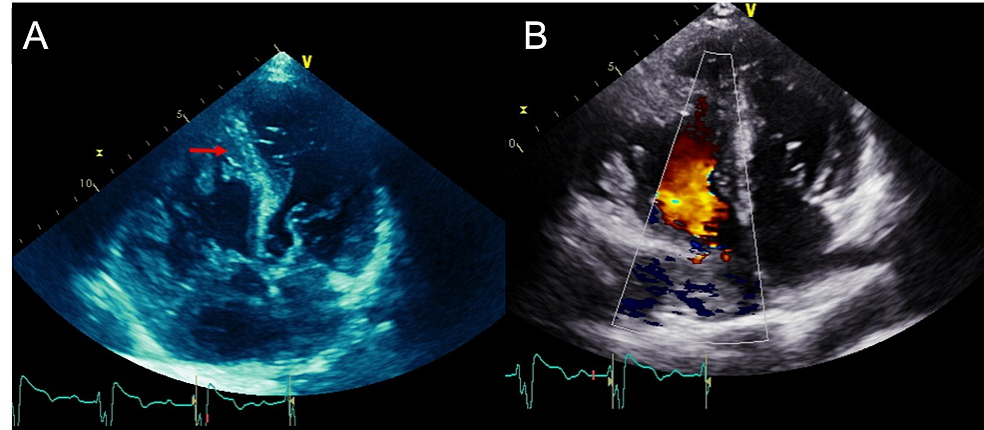
Two-dimensional (A) and color doppler (B) four-chamber views demonstrate a trabeculated right ventricle (arrow).

A 66-year-old male with a past medical history of hypertension, stroke, and ventral septal defect requiring surgical repair presented to the emergency department with a complaint of substernal and left-sided chest pain. The chest pain, which was provoked by activity, had been present for several months with each episode typically lasting only for a few seconds. However, over the last week, episodes had gradually worsened and lasted for 15 to 20 minutes at a time. Electrocardiogram (ECG) in the emergency department demonstrated sinus rhythm with wide QRS complexes and deeply inverted T waves. Cardiology was consulted for this patient and a transthoracic echocardiogram was performed (Figure 1).

**Figure 2:**
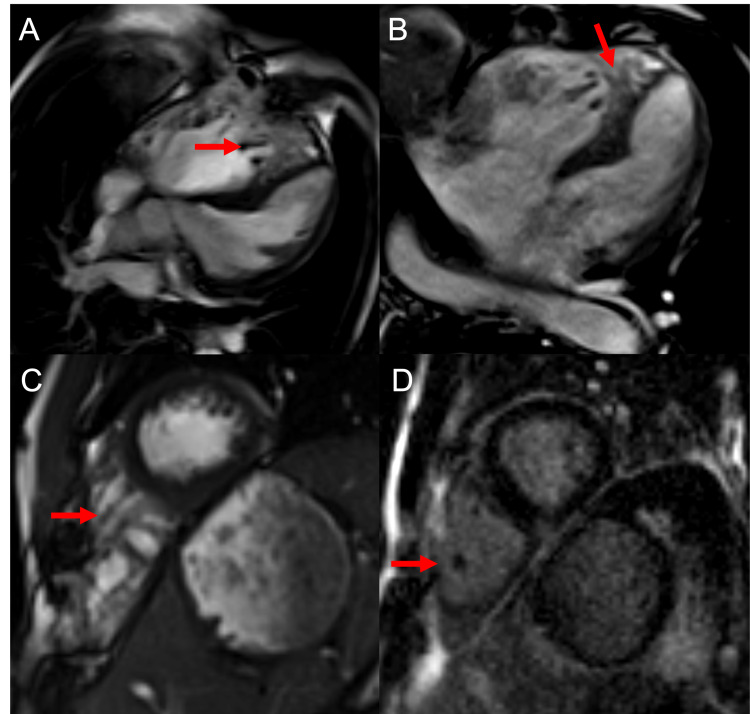
(A-C) Balanced steady-state free precession four-chamber and short-axis views demonstrating heavily noncompacted right ventricle without significant free wall thickening (arrows). (D) Post-contrast delayed gadolinium enhancement image does not show abnormal enhancement of the myocardium in the area of trabeculations (arrow) to suggest fibrosis. There is no evidence for cardiac thrombus.

The echocardiogram was difficult to perform as the patient had underlying scoliosis, which limited the operator's ability to fully assess the heart. The ejection fraction was 50-55%, the ventricular septum was hypertrophied, and a patch along the membranous portion of the ventricular septum without residual shunt was seen consistent with a reported history of surgical repair. The right heart was difficult to assess, but the right ventricle appeared markedly enlarged with diffuse trabeculations and hypokinesis. The cardiologist team was concerned for noncompaction of the right ventricle or arrhythmogenic right ventricular dysplasia (ARVD). Cardiac MRI was requested for further evaluation (Figure 2).

Cardiac MRI demonstrated a thickened septal wall with patch repair consistent with past surgery and an enlarged right ventricle with global hypokinesis, corroborating the findings on the echocardiogram. The right ventricular trabeculae extending towards the cardiac apex demonstrated a thickened appearance measuring up to 3.4 cm. There was no delayed enhancement to suggest fibrosis nor area of focal dilatation, which are key features that exclude ARVD. In addition, tricuspid valve annular motion was normal, and no regional wall motion abnormalities were identified, which also excluded ARVD as a potential diagnosis. A diagnosis of right ventricular noncompaction was made. The patient would be optimized medically and discharged in stable condition with appointments to follow-up in the outpatient cardiology clinics.

## Discussion

Isolated right ventricular noncompaction has been sparsely described in the literature. Reports have been published where exploration was prompted by symptoms of right-sided heart failure [11-13]. A diagnostic workup of right ventricular noncompaction has also been performed following ECG ST-segment elevation in leads V1-V3 [14]. No specific set of diagnostic criteria has been established for practical use in diagnosing right ventricular noncompaction and previously reported findings on doppler echocardiography of left ventricular noncompaction are utilized in published cases of right-sided disease [12,14,15]. Echocardiographic findings include segmental thickening of the myocardial with two layers and prominent trabeculae with a thin epicardial and thickened endocardial layer [15]. Echocardiographic evaluation of our patient with these previously mentioned criteria was used but was limited due to patient scoliosis. It was decided that a multi-modality approach would be best in effectively describing his condition.

MRI has been used as an effective means of diagnosing left ventricular noncompaction and it provides an adequate correlation of findings from echocardiography [8,16]. It is also useful in cases that have poor or limited echocardiographic images, which was the case with our patient [8]. T2-weighted and contrast-enhanced images allow for better discrimination between compact and noncompact inner and outer layers of the myocardium [16]. The signal intensity of MRI can also be used to identify damaged areas of the myocardium that may be a nidus for fatal arrhythmias [8,16]. In our case, MRI was used to rule out the presence of ARVD. The right ventricular free wall was not abnormally thickened but rather there was an abundance of noncompacted trabeculations. MRI has also been shown to effectively diagnose cases of left ventricular noncompaction based on a ratio of compacted to noncompacted myocardium; a diastolic ratio of >2.3 had a sensitivity and specificity of 86% and 99%, respectively for diagnosing the condition [17]. However, this ratio is not well applied to the right ventricle as there are inherent differences between right and left heart anatomy and physiology. In addition, cardiac MRI may be used to allow the clinician to effectively stage the condition by interpreting the degree of trabecular delayed hyperenhancement on MRI with a 16-segment model [18]. 

Although the majority of data utilizes MRI in diagnosing left ventricular noncompaction, this does not discount its utility in diagnosing right ventricular noncompaction. In our case, a multi-modality approach worked best for ultimately diagnosing the condition. MRI was needed in order to rule out arrhythmogenic right ventricular dysplasia. A small study in 2003 concluded that echocardiography represented the standard diagnostic modality for ventricular noncompaction compared to the MRI [17,19]. However, advances in MRI have drastically improved image quality and increased diagnostic accuracy [19]. Future studies are needed to better elucidate clear diagnostic criteria of right ventricular noncompaction and establish a multimodality graded protocol based on patient presentation. 

## Conclusions

Although no definitive diagnostic criteria exist for right ventricular noncompaction, the criteria for left ventricular noncompaction can be used as a good benchmark. Diagnosis of right ventricular noncompaction and exclusion of other cardiomyopathies can be achieved using multi-modality imaging and a proper review of the clinical history.
